# Rho Kinase Regulates Aortic Vascular Smooth Muscle Cell Stiffness Via Actin/SRF/Myocardin in Hypertension

**DOI:** 10.1159/000485284

**Published:** 2017-11-23

**Authors:** Ning Zhou, Jia-Jye Lee, Shaunrick Stoll, Ben Ma, Kevin D. Costa, Hongyu Qiu

**Affiliations:** a Division of Cardiology, Department of Internal Medicine, Tongji Hospital, Tongji Medical College, Huazhong University of Science and Technology, China; b Division of Physiology, Department of Basic Sciences, School of Medicine, Loma Linda University, Loma Linda, CA, USA; c Cardiovascular Research Center, Icahn School of Medicine at Mount Sinai, New York, NY, USA

**Keywords:** Aortic stiffness, Atomic force microscopy, Hypertension, Rho kinase, Vascular smooth muscle cell, Serum response factor, Myocardin

## Abstract

**Background/Aims:**

Our previous studies demonstrated that intrinsic aortic smooth muscle cell (VSMC) stiffening plays a pivotal role in aortic stiffening in aging and hypertension. However, the underlying molecular mechanisms remain largely unknown. We here hypothesized that Rho kinase (ROCK) acts as a novel mediator that regulates intrinsic VSMC mechanical properties through the serum response factor (SRF)/myocardin pathway and consequently regulates aortic stiffness and blood pressure in hypertension.

**Methods:**

Four-month old male spontaneously hypertensive rats (SHR) and Wistar-Kyoto (WKY) rats were studied. Aortic stiffness was measured by echography. Intrinsic mechanical properties of VSMCs were measured by atomic force microscopy (AFM) *in vitro*.

**Results:**

Compared to WKY rats, SHR showed a significant increase in aortic stiffness and blood pressure, which is accompanied by a remarkable cell stiffening and ROCK activation in thoracic aortic (TA) VSMCs. Theses alterations in SHR were abolished by Y-27632, a specific inhibitor of ROCK. Additionally, boosted filamentous/globular actin ratio was detected in TA VSMCs from SHRversus WKY rats, resulting in an up-regulation of SRF and myocardin expression and its downstream stiffness-associated genes including α-smooth muscle actin, SM22, smoothelin and myosin heavy chain 11. Reciprocally, these alterations in SHR TA VSMCs were also suppressed by Y-27632. Furthermore, a specific inhibitor of SRF/myocardin, CCG-100602, showed a similar effect to Y-27632 in SHR in both TA VSMCs stiffness *in vitro* and aorta wall stiffness *in vivo*.

**Conclusion:**

ROCK is a novel mediator modulating aortic VSMC stiffness through SRF/myocardin signaling which offers a therapeutic target to reduce aortic stiffening in hypertension.

## Introduction

Hypertension is a highly age-related disease and affects more than 1 billion people worldwide. The progressive increase in blood pressure (BP) with age is characterized by a greater increase in isolated systolic hypertension, a larger elevation in systolic blood pressure (SBP) than diastolic blood pressure (DBP), leading to an accelerating rise in pulse pressure (PP) after 50 years [1–3]. Although it is widely accepted that the increase in SBP with advancing age is mostly consistent with the large artery stiffening [2, 4–7], there is still no consensus on what are the primary causes of these disorders. Our recent studies show that increased intrinsic stiffness of vascular smooth muscle cells (VSMCs) in aorta is an important contributor to the pathogenesis of aortic stiffening in both aging and hypertension, and that this could be a novel target for future anti-aortic stiffness drug development [8–10]. However, less is known about molecular regulation involved in the VSMC stiffening in large arteries.

Rho kinase (Rho-associated coiled-coil-containing protein kinase, hereafter referred to as ROCK) is a serine/threonine protein kinase that contains an N-terminal catalytic kinase domain, which has been identified as one of the effector of the small GTP-binding protein Rho. Although accumulating evidences have demonstrated that the ROCK pathway plays a crucial role in the pathogenesis of hypertension [8, 11, 12], ROCK has not previously been shown to be involved in cellular stiffening of VSMC. Inhibition of ROCK with Y-27632, fasudil or SAR407899 significantly reduced blood pressure in human and animal models of hypertension [12–15], despite the precise molecular mechanism underling the antihypertensive effect not being fully understood. In both experimental and clinical studies, ROCK has been demonstrated to enhance the phosphorylation of myosin light chain (MLC) and mediate actin cytoskeletal organization and adhesion [16, 17]. Considering the important role of the MLC kinase (MLCK) and cytoskeletal and adhesion proteins in VSMC stiffness presented in previous studies [9, 18], we expect there to be a link between this pathway and VSMC stiffness. Thus, we hypothesize that ROCK participates in the regulation of aortic stiffness via altering VSMC stiffness in hypertension. Therefore, in this study, we integrated atomic force microscopy (AFM) and molecular approaches to determine whether increased stiffness of aortic VSMCs in hypertensive rats is ROCK-dependent, and whether the anti-hypertensive effect of ROCK inhibitors contributes to the reduction of aortic stiffness via changing VSMC mechanical properties. In addition, our most recent study demonstrated that serum response factor (SRF)/myocardin pathway acts as a pivotal mediator of aortic VSMC stiffening and plays a central role in the pathological aortic stiffening in hypertension [10]. Therefore, we further investigated interlink between the ROCK activation and SRF/myocardin pathway in the regulation of aortic VSMC stiffness in the present study.

## Materials and Methods

### Animal model

Adult (4 month-old) male spontaneously hypertensive rats (SHR) and normotensive control Wistar-Kyoto (WKY) rats (Charles River Laboratories, San Diego, CA) were studied as described previously [10]. All animal procedures were performed in accordance with NIH guidelines (Guide for the Care and Use of Laboratory Animals, revised 2011) and protocols approved by the Institutional Animal Care and Use Committee of Loma Linda University. For *in vivo* drug treatments, Y-27632 (0.3 mg/kg/h, ApexBio Technology, TX), CCG-100602 (7.5 mg/kg/d, ApexBio Technology, TX) or vehicle control (DMSO, Sigma-Aldrich) were continuously administered for 2 weeks by Alzet osmotic minipumps (Model 2ML2, DURECT, CA), implanted subcutaneously in rats under anesthesia with 2% isoflurane (JD Medical, AZ) [10].

### Measurement of blood pressure

Systemic systolic and diastolic blood pressures (SBP and DBP) were measured in conscious animals by restraint tail cuff every two days for 2 weeks using the CODA system (Kent Scientific, CT) as described previously [10]. Aortic blood pressure (ABP) was evaluated as previously described [19]. A catheter (Millar 2.0 F, model SPR 320, Millar Instruments, Inc., Houston, TX) was inserted via the right common carotid artery into aorta and carefully introduced into the aortic root under anesthesia with an inspired 2% isoflurane (JD Medical, AZ). The transducer was connected to Power Laboratory system (AD Instruments, Castle Hill, Australia). Systolic and diastolic aortic pressure (SAP and DAP) were recorded [10]. Pulse pressure (PP) was calculated using the formula: PP = SAP-DAP.

### Measurement of aortic stiffness in vivo

Hemodynamic assessment was performed by doppler ultrasound echocardiography under anesthesia with 2% isoflurane (JD Medical, AZ) simultaneously with the non-invasive tail-cuff (baseline and 1 week) or invasive catheter (2 week) BP measurement. The following measurements were performed: heart rate (HR), cardiac output (CO), diastolic diameter of the thoracic aorta (D), systolic minus diastolic diameter change (ΔD). Regional aortic stiffness was evaluated by arterial compliance (C) which is the absolute change in diameter (ΔD) for a given pressure step (PP) (C =ΔD/PP) and arterial strain (ΔD/D) [10].

### VSMC isolation, culture and treatments

Rats were euthanized with carbon dioxide inhalation and artery tissues were rapidly collected. Primary VSMCs were isolated from aorta and arteries of SHR and WKY rats and serially cultured for up to three to four passages as described previously [10, 20]. VSMCs were treated with Y-27632 (10 μmol/L) or CCG-100602 (25 μmol/L) for 24 hours and then were collected for RNA and protein extraction or prepared for immunostaining. DMSO was used as a vehicle control.

### VSMC stiffness measured by atomic force microscopy (AFM)

Single-cell micromechanical measurements were performed using a biological AFM system (Asylum Research, MFP-3D-BIO, CA) with a silicon nitride AFM probe (nominal spring constant, k = 0.1 N/m) with a pyramidal tip (radius 40 nm). As we recently described [10], two nanoindentation protocols were used to determine the cellular micromechanics: (1) spatial variation, which indented multiple locations per cell between the nucleus and periphery to examine mechanical heterogeneity, and (2) temporal variation, which repeatedly indented one site every 10 seconds for 30 minutes to assess spontaneous changes in local VSMC mechanical properties. The apparent elastic modulus (E_ap_) was determined using Hertz contact analysis for a cone to model the indentation force curve. The effects of drug interventions on VSMC stiffness were also assessed. Isolated VSMCs in subconfluent monolayer culture were treated for 24 hours with Y-27632 (22.5 to 2250 nmol/L), or CCG-100602 (1.12 μmol/L) or vehicle control (DMSO) prior to AFM indentation testing as described above.

### RNA extraction and real-time PCR

RNA was extracted from isolated VSMCs by using Quick-RNA MiniPrep kit (Genesee Scientific, Cat No. 11–327) according to the manufacturer’s instructions. Quantitative real-time PCR was performed on a CFX96 Touch™ Real-Time PCR Detection System by using iTaq™ Universal SYBR® Green Supermix (BioRad, Cat No. 1725121) according to the manufacturer’s instructions. All real-time PCRs were performed in triplicate as described in our previous study [10, 21].

### Protein extraction and Western blot

Total protein was extracted from VSMCs using cell extraction buffer (Life Technologies, Cat No. FNN0011) as described previously [9, 10, 22]. Subcellular fractions were extracted using the Nuclear Extraction Kit (Millipore Inc., USA). Protein expression levels were quantified by Western blotting as described previously [10, 23] and were detected using the LI-COR Odyssey® Infrared Imaging System (LI-COR Biosciences, Lincoln, NE). HDAC1 and GAPDH were used as loading controls for nuclear fraction and total cell lysates, respectively [10].

### Rho-kinase activity

Activity of ROCK was measured by using a ROCK activity assay kit (Millipore, CSA001) according to the manufacturer’s instructions.

### Immunostaining

VSMCs were fixed with 4% paraformaldehyde in PBS, permeabilized in 0.2% Triton X-100 and then stained with primary α-SMA antibody (Sigma) at a dilution of 1:100 using standard immunofluorescence staining techniques as described previously [9, 24].

### F-actin/G-actin measurements

The F/G-actin ratio in VSMCs was determined by Western blotting using the G-actin/F-actin *in vivo* assay kit (Cytoskeleton Inc. BK037, CO) [25] and by immunostaining using Alexa Fluor® 568 Phalloidin (ThermoFisher Scientific, A12380, MA) and Deoxyribonuclease I, Alexa Fluor® 488 Conjugate (Life Technologies, A10042, MA) [26].

### Statistical analysis

Results are presented as the mean ± SEM for the number of samples indicated in the figure legends. One-way ANOVA or two-way ANOVA was used to test effects of group (WKY vs. SHR), region (TA vs. RA), and drug intervention. Student-Newman-Keuls post hoc correction was applied for multiple pairwise comparisons. A value of p<0.05 was considered statistically significant.

## Results

### Inhibition of ROCK by Y-27632 reduced aortic stiffness and induced a disproportional reduction in SBP in SHR

**Fig. 1.**
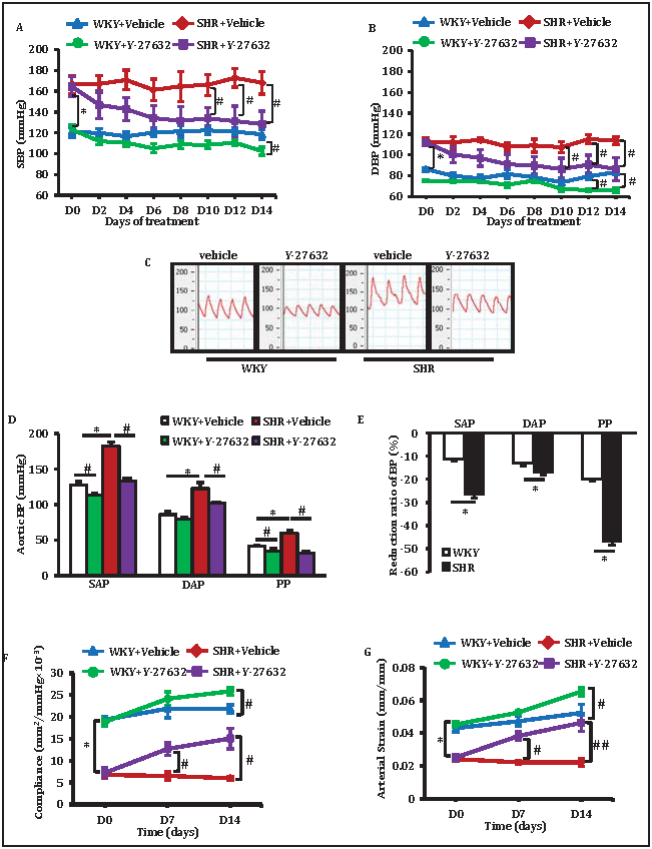
Effects of Y-27632 on the blood pressure and aortic stiffness in SHR and WKY rats. (A-B) Time dependent changes of systolic blood pressure (SBP) (A) and diastolic blood pressure (DBP) (B) measured by tail cuff at conscious status. (C) Representative waveform of aortic blood pressure measured invasively by a Millar catheter at the end of two-week treatment. (D) Systolic Aortic blood pressure (SAP), diastolic blood pressure (DAP) and pulse pressure (PP) under the treatments. (E) Reduction rate of blood pressure versus vehicle control. (F-G) Aortic stiffness in SHR and WKY rats in vivo evaluated by echography before (D0) or at day 7 (D7) and day 14 (D14) after the initiation of the treatments with Y-27632 or vehicle (DMSO), reflected by the change of arterial com-pliance (F) and arterial strain (G). Data are shown as mean ± SEM, *P<0.01 vs. corresponding WKY. ^#^P<0.05, ^##^P< 0.01 vs corresponding vehicle. n= 5 rats/group.

Both SHR and WKY rats were treated with chronic delivery of Y-27632 or vehicle for 2 weeks. Systemic blood pressure was continuously traced every two days by tail cuff in conscious animals. With the treatment of vehicle, SBP and DBP of SHR were significantly higher compared to WKY rats. Y-27632 treatment significantly reduced SBP and DBP in SHR compared to vehicle, starting at the 10^th^ day of treatment, while it modestly reduced SBP and DBP in WKY rats at the end of day 14 and 12 of the treatment, respectively (Fig. 1A and 1B). To confirm the non-invasive BP measurements, direct aortic pressure was measured by an invasive catheter at the end of two-week treatment of Y-27632 or vehicle (Fig. 1C and 1D). Consistent with tail cuff findings, systolic aortic pressure (SAP) and diastolic aortic pressure (DAP) were significantly higher in SHR compared to WKY rats with vehicle treatment, resulting in a greater pulse pressure (PP). Y-27632 treatment induced a remarkable reduction of SAP and DAP in SHR, and a slight reduction of SAP in WKY rats at the end of 2 weeks treatment compared to the vehicle groups (Fig. 1D). Additionally, Y-27632 resulted in a larger reduction of SAP (27%) than DAP (17%), which led to a significant reduction of PP by 47% in SHR (Fig. 1E). These observations indicated that Y-27632 has a prominent effect on SAP versus DAP, implying a potential effect on aortic relaxation through the reduction of aortic stiffness. Moreover, Y-27632 was more effective in SHR than in WKY as indicated by the significantly greater reduction of blood pressure in SHR than in WKY rats in terms of SAP, DAP and PP (Fig. 1E).

We next tested whether the reduction of BP could be attributed to the reduction of aortic wall stiffness. Using echography, significantly increased local aortic wall stiffness was detected in SHR compared to WKY rats with the vehicle treatment, evidenced by lower arterial compliance (Fig. 1F) and arterial strain (Fig. 1G). Compared to vehicle, Y-27632 treatment significantly reduced the aortic stiffness in SHR as early as at day 7 post the initial treatment and further neutralized the stiffness at day 14. Y-27632 showed a weaker and delayed effect on aortic stiffness in WKY rats compared to SHR (on treatment day 14) (Fig. 1F and 1G). Notably, the reduction of aortic wall stiffness preceded the decrease of blood pressure by about 3 days. These data indicated that Y-27632 reduces aortic stiffness, which may serve as a primary mechanism of its disproportionate effects on reducing SBP and SAP in SHR.

### Amelioration of aortic VSMC stiffening contributed to the reduction of aortic stiffness by Y-27632

**Fig. 2.**
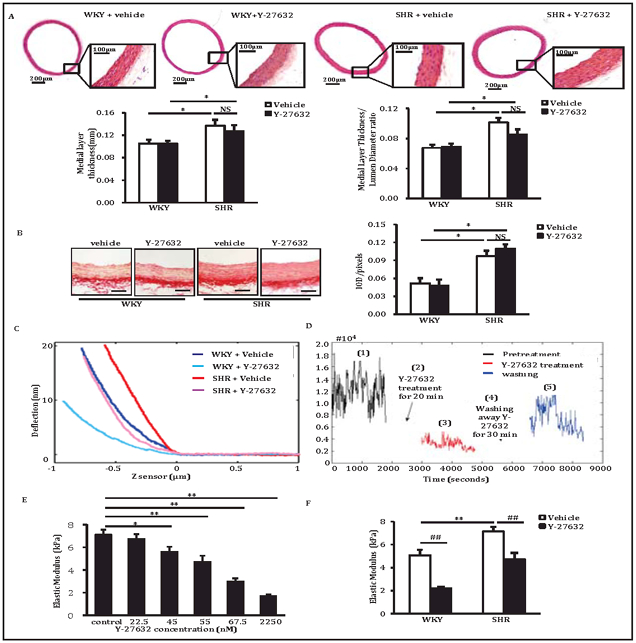
Effect of Y-27632 on TA VSMC stiffness. (A) Thickness of medial layer of aortic wall and the ratio of lumen diameter. (B) Collagen deposition in medial layer of aortic wall. Scale bar: 100μm, n = 5 rats/group. (C) Representative force curves during advance from atomic force microscopy (AFM) nanoindentation of vascular smooth muscle cells (VSMCs) from the thoracic aorta (TA). (D) Representative of the reversible changes of elastic modulus with or without the presence of Y-27632 in SHR TA VSMC. (E) Representative dose response of SHR TA VSMC stiffness to Y-27632. (F) Average apparent elastic modulus of TA VSMCs. n= 4 rats/group. Data are shown as mean ± SEM, *P<0.01 vs. corresponding WKY. ^#^P<0.05, ^##^P< 0.01 vs corresponding vehicle. NS: no significant difference.

To determine the potential contributors to the reduction of aortic stiffness by Y-27632 described above, the alterations of vascular wall thickness, collagen deposition and VSMC mechanical properties were measured. Significantly increased medial layer thickness of aortic wall, the ratio of medial thickness and aortic lumen diameter, and total density of collagen were observed in vehicle treated SHR compared to WKY rats (Fig. 2A and 2B). However, the above indexes of aortic remodeling showed no remarkable alterations during the two-week treatment with Y-27632 compared to vehicle, despite the attenuation of aortic stiffening, indicating that the anti-stiffening effects of Y-27632 on the aorta could not be attributed to the reversal of aortic wall remodeling during this short-term intervention.

We then measured the alteration of intrinsic stiffness of individual VSMCs by using AFM. As shown in Fig. 2C, a steeper advancing curve was observed in vehicle-treated TA VSMCs from SHR compared with WKY rats, indicating increased cell stiffness in SHR TA VSMCs. Y-27632 treatment reduced the TA VSMC stiffness in SHR to a level similar to that in untreated WKY Moreover, we observed a reversible effect of Y-27632 on VSMC stiffness during AFM measurement of temporal variations. As shown in Fig. 2D, following a 30-minute period of repeated indentations at baseline (step 1), Y-27632 treatment of SHR TA VSMCs for 20 minutes (step 2) substantially reduced VSMC E_ap_ (step 3); After washing away Y-27632 for 30 minutes (step 4), the VSMC elastic modulus began to return back toward baseline (step 5). This data indicated that the reduction of VSMC stiffness specifically relied on the inhibition of ROCK by Y-27632 (Fig. 2D). To examine the sensitivity of VSMC stiffness to Y-27632 treatment, TA VSMCs from SHR rats were treated for 24 hours with different concentrations of Y-27632, a significant dose-dependent reduction of VSMC stiffness (E_ap_) was observed with the concentrations of Y-27632 from 22.5 nM to 2.25 μM (Fig. 2E). Further comparison of the effect of Y-27632 on VSMC stiffness from SHR and WKY rats was performed on TA VSMCs treated with a selected concentration of 55 nM of Y-27632 or with vehicle. As shown in Fig. 2F, the average local E_ap_ of TA VSMCs was significantly higher in SHR (7.1± 0.1 kPa) vs. WKY rats (5.1 ± 0.1 kPa) (*P*<0.01) under vehicle control conditions. Treatment with Y-27632 reduced the TA VSMC stiffness in both SHR and WKY compared with the respective vehicle controls (*P*<0.01 vs. vehicle). These findings confirmed the temporal measurements.

### ROCK activity was increased in stiffened TA VSMCs but preserved in non-stiffened RA VSMCs

**Fig. 3.**
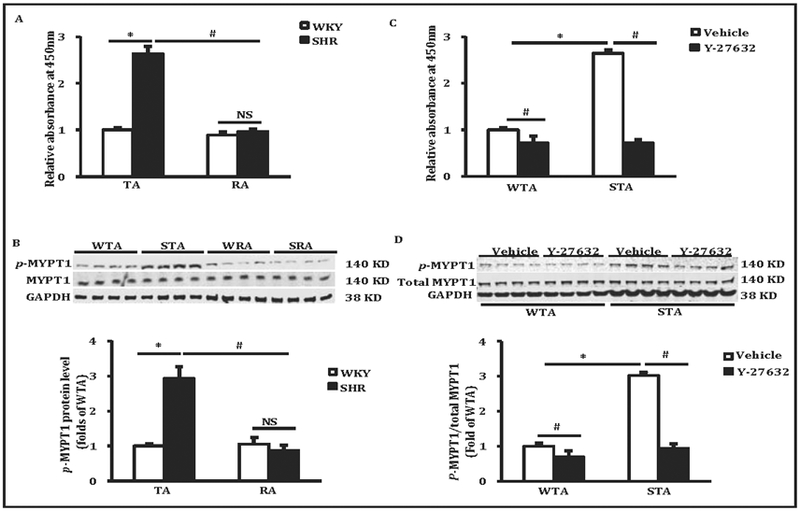
The ROCK activity in TA VSMCs. (A) The Rho kinase (ROCK) activity of VSMCs in thoracic aorta (TA) and renal artery (RA) detected by ROCK activity kit. (B) Western blots and the quantified data of phosphorylation of myosin phosphatase target subunit 1 (MYPT1) (Thr696) in TA and RA. *P<0.01 vs WKY treated with vehicle, ^#^P< 0.01 vs corresponding TA. (C) The ROCK activity of TA VSMCs under the treatment of Y-27632 or vehicle detected by ROCK activity kit. (D) Effect of Y-27632 on phosphorylation of MYPT1 (Thr696) in TA. GAPDH was used as loading control. Data are shown as mean ± SEM, *P<0.05 vs WKY, ^#^P<0.05 vs corresponding vehicle. NS: no significant difference.

**Fig. 4.**
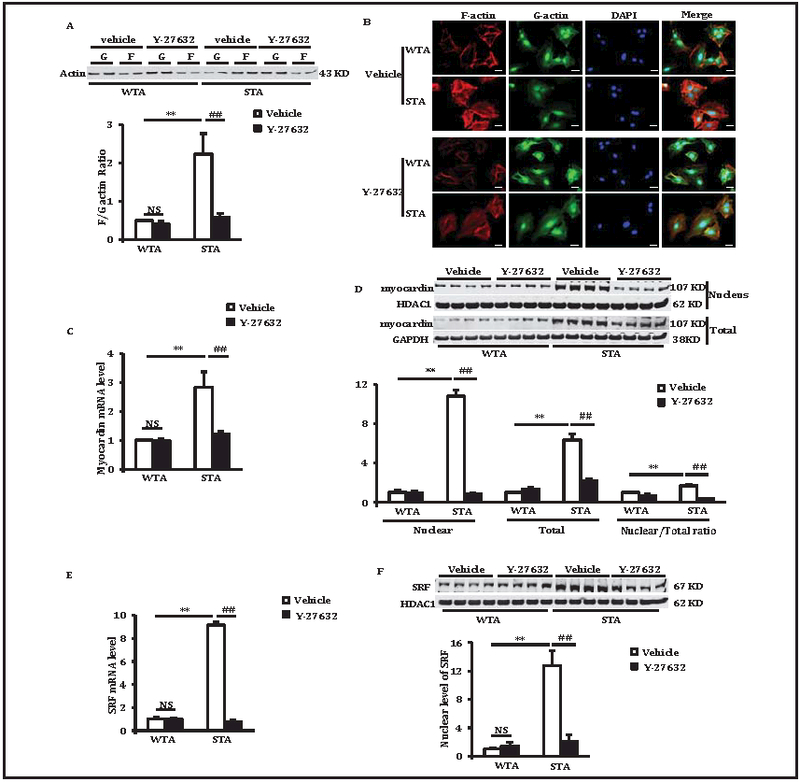
Effect of Y-27632 on actin/SRF/myocardin pathway in TA VSMCs. (A) Effects of Y-27632 on ratio of F/G actin detected by western blotting. (B) Fluoresce staining of F- and G-actin in primary WKY rat thoracic aorta (WTA) and SHR thoracic aorta (STA) VSMCs. Scale bar: 50μm. (C) The mRNA level of myocardin in WTA and STA VSMCs. (D) Representative western blots and the quantified data of myocardin in nuclear fractions and total cell lysates from WTA and STA VSMCs and the ratio of nuclear/total protein. (E) The mRNA level of SRF in WTA and STA VSMCs. (F) Representative Western blots and the quantified data of SRF in nuclear fractions from WTA and STA VSMCs. GAPDH and HDAC1 were used as loading control of total cell lysates and nuclear fractions, respectively. Data are shown as mean ± SEM, **P<0.01 vs WTA, ^##^P<0.01 vs corresponding vehicle. NS: no significant difference.

**Fig. 5.**
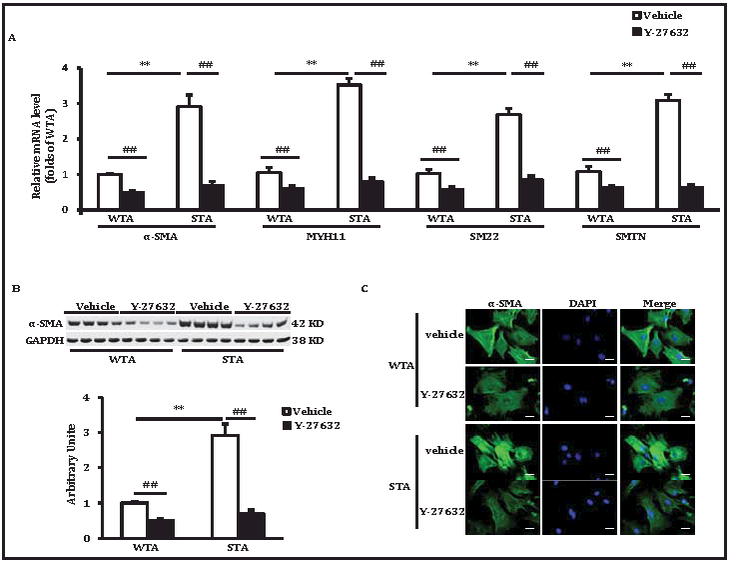
Effects of Y-27632 on the expression of stiff-ness-associated genes in VSMCs. (A) The relative mRNA levels of SRF/myocardin target genes in VSMCs, α-SMA: al pha-smooth muscle ac-tin; MYH11: myosin heavy chain 11; SM22: smooth muscle 22; SMTN: smoothelin. (B) Represen-tative western blots and the quantified data of α-SMA in total cell lysates. GAPDH was used as loading control. (C) Immunostainings of α-SMA in VSMCs. Scale bar: 50μm. Data are shown as mean ± SEM, **P<0.01 vs WTA, ^##^P<0.01 vs corresponding vehicle. All, n=5 rats/group.

**Fig. 6.**
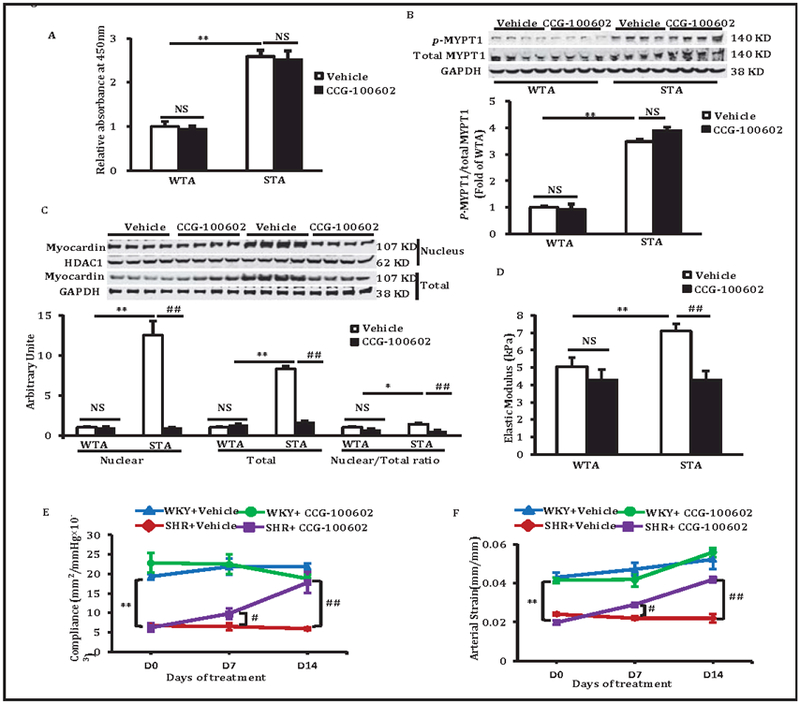
Effect of the inhibition of SRF/myocardin by CCG-100602. (A) The ROCK activity of TA VSMCs detected by ROCK activity kit. (B) Representative western blots and the quantified data of ROCK substrate, phosphorylation of MYPT1 (Thr696) in TA VSMCs. (C). Representative western blots and the quantified data of myocardin in nuclear fractions and total cell lysates and their ratio. (D) TA VSMC stiffness detected by AFM. (E and F) Aortic stiffness in SHR and WKY rats evaluated by echography before (D0) or at day 7 (D7) and day 14 (D14) after the initiation of the treatments with CCG-100602 or vehicle (DMSO) reflected by the change of arterial compliance (E) and arterial strain (F). Data are shown as mean ± SEM, *P<0.05, **P<0.01 vs corresponding WKY, ^#^P<0.05, ^##^P<0.01 vs vs corresponding vehicle.

**Fig. 7.**
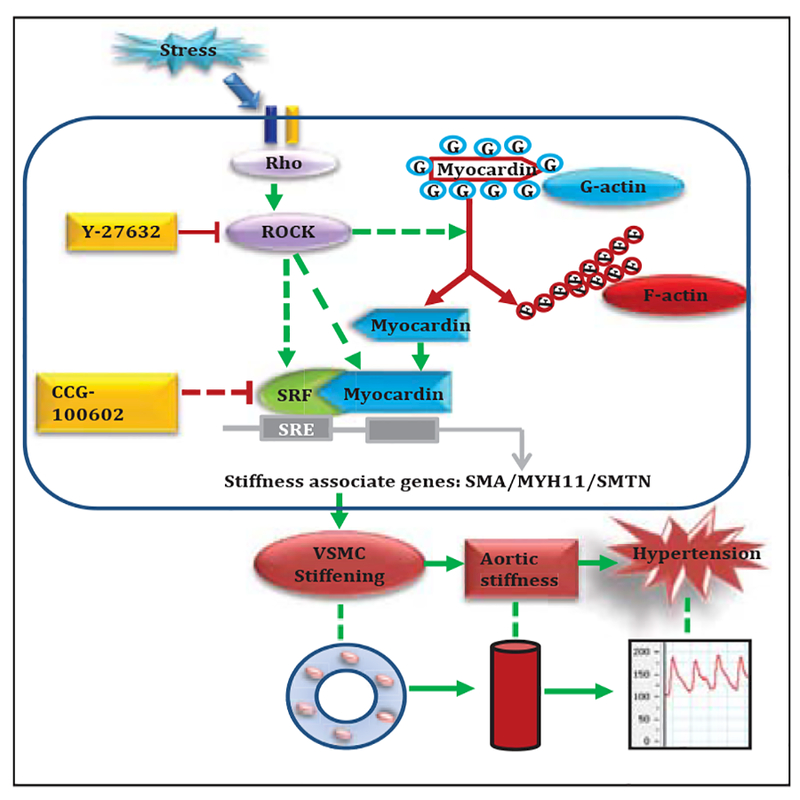
An illustration of the mechanism. SRF/myocardin pathway acts as an essential regulator of VSMC stiffness and plays a central role in the aortic stiffness associated with hypertension.

To determine the role of ROCK in the regulation of intrinsic TA VSMC stiffness, two complementary approaches were used: first, we tested whether ROCK activity was specifically increased in aortic VSMCs, and second, we tested whether Y-27632 inhibited ROCK activity selectively in aortic VSMCs. Our previous studies showed that stiffness was significantly increased in VSMCs isolated from TA but not in RA from the same SHR rat [10], this regional difference provided an ideal cell model to test the specific molecular changes associated with aortic stiffness. To examine the molecular mechanism underlying the effect of Y-27632 on VSMC stiffness described above, ROCK activity was tested in isolated VSMCs from both TA (stiffened VSMCs) and RA (non-stiffened VSMCs) in SHR using a ROCK activity assay kit, and compared to WKY rats. As shown in Fig. 3A, ROCK activity increased nearly 3-fold in VSMCs from SHR TA compared to WKY TA, whereas no difference was observed in RA. These observations were further confirmed by Western blot (Fig. 3B) using a primary antibody against phosphorylated myosin phosphatase target subunit 1(MYPT1), a specific substrate of ROCK. Together, these results indicate a correlation between ROCK activity and VSMC stiffening. To test whether Y-27632-induced correction of stiffening resulted from a direct inhibition of ROCK activity in VSMCs, isolated TA VSMCs were treated with Y-27632 and compared to vehicle-treated controls. As shown in Figures 3C and 3D, Y-27632 significantly abrogated the ROCK activity in TA VSMCs from both SHR and WKY rats, indicating an inhibitory effect of Y-27632 on ROCK activity in VSMCs. Taken together, a higher ROCK activity in TA VSMCs parallels the elevated cell stiffness, which can be reversed by Y-27632.

### ROCK regulated actin/SRF/myocardin pathway in stiffening TA VSMCs

Next we explored the signaling pathway involved in the regulation of VSMC stiffness by ROCK in hypertension. We first tested the content of filamentous (F) and globular (G) actin in VSMCs treated with Y-27632 or vehicle, using western blot and specific immunostaining. Compared to WKY rats, the F/G-actin ratio was strikingly increased by 2.2-fold in vehicle-treated TA VSMCs from SHR, which was almost completely blocked by treatment with Y-27632 (Fig. 4A). This observation was further confirmed by specific immunostaining of F- and G-actin in individual isolated VSMCs using phalloidin and deoxyribonuclease I, respectively (Fig. 4B). Consistent with the shift of the F/G-actin ratio, we also found that myocardin, a master regulator of smooth muscle gene expression in VSMCs, was strikingly increased at the mRNA level in stiffened TA VSMCs in SHR (Fig. 4C). In addition, a parallel increase of nuclear distribution of myocardin was observed in SHR TA VSMCs versus WKY TA VSMCs. Compared to WKY, TA VSMCs from SHR showed an increase of myocardin by 10.7-fold in nuclear fractions and 6.3-fold in total cell lysates, resulting in an increased ratio of nuclear/total myocardin expression by 1.7-fold (Fig. 4D). Treatment with Y-27632 restored the nuclear expression of myocardin to a level observed in WKY VSMCs (Fig. 4D), which resulted in lowering the F/G-actin ratio (Fig. 4A and 4B), indicating a link between ROCK-regulated F/G-actin balance and the myocardin nuclear distribution. Moreover, we detected a significant increase in the expression of serum response factor (SRF), a key transcriptional factor and cofactor of myocardin, in SHR TA VSMCs compared to WKY at both the mRNA (Fig. 4E) and the protein (Fig. 4F) levels in the cellular nuclear fraction by 9.1- and 12.7-fold, respectively. Furthermore, the upregulation of SRF was essentially blocked by treatment with Y-27632 (Fig. 4E and 4F).

### Inhibition of ROCK reduced expression of SRF/myocardin downstream target genes in TA VSMCs

To evaluate whether the alteration of SRF/myocardin resulted in reprogramming of the downstream target genes, we observed strikingly up-regulated mRNA expression of alpha--smooth muscle actin (α-SMA), myosin heavy chain 11 (MYH11), smooth muscle 22 (SM22) and smoothelin (SMTN) in TA VSMCs from SHR compared to WKY rats (all *P*<0.01); these elevated gene expression levels were rectified by treatment with Y-27632 (Fig. 5A). Western blotting was performed to further confirm the alteration of corresponding protein levels of these genes. As shown in Fig. 5B, protein expression of α-SMA was significantly increased in TA VSMCs from SHR compared with WKY by 2.9-fold. Y-27632 treatment significantly decreased α-SMA protein level by 76% and 50% in SHR and WT TA VSMCs, respectively (Fig. 5B). These results were further confirmed by the findings of α-SMA immunostaining in individual VSMCs (Fig. 5C).

### Inhibition of SRF/myocardin ameliorated the stiffness of aortic VSMCs and aortic wall

Our data presented above indicated that SRF/myocardin acts as a linker between ROCK and VSMC stiffness. To test whether SRF/myocardin are necessary for ROCK mediated VSMC stiffness, a specific inhibitor of SRF/myocardin interaction, CCG-100602, was used to block the SRF/myocardin interaction. We found that treatment with CCG-100602 did not change ROCK activation in TA VSMCs from SHR compared to vehicle, indicating that CCG-100602 targets the downstream pathway of ROCK (Fig. 6A and 6B). However, similar to the effect of Y-27632, treatment with CCG-100602 significantly attenuated the increase of myocardin nuclear distribution (Fig. 6C). In addition, by using a temporal indentation, we found that CCG-100602 attenuated VSMC stiffening in SHR TA VSMCs from continuous measurement, which is consistent with our recent studies with spatial indentations of AFM [10] (Fig. 6D). Interestingly, these effects of CCG-100602 were not observed in WKY TA VSMCs, indicating that CCG-100602 selectively corrected pathological alterations of VSMCs in the hypertensive aorta (Fig. 6D), and as we have recently studied in detail [10].

To test whether the reduction of VSMC stiffness by the inhibition of SRF/myocardin with CCG-100602 restrained the aortic stiffness *in vivo*, both SHR and WKY rats were administered with CCG-100602 or vehicle for 2 weeks. Arterial compliance and strain were measured using Doppler ultrasound echocardiography. As shown in Fig. 6E and F, treatment with CCG-100602 abrogated the increase of aortic stiffness represented by reduced arterial compliance and strain, indicating a significant anti-stiffening effect resulting from the inhibition of SRF/myocardin. Consistent with the observations on aortic VSMC stiffness *in vitro*, CCG-100602 has no significant effects on aortic wall stiffness in WKY rats. Taken together, inhibition of SRF/myocardin can ameliorate the VSMC stiffness led by activated ROCK.

## Discussion

Aortic stiffening is a hallmark of aging. Emerging evidence has been demonstrated that increased aortic stiffness is not only a fundamental manifestation of vascular aging, but also an independent predictors for incident hypertension [27]. It has been also reported that aortic stiffening is a harbinger of other age-associated diseases such as stroke, myocardial infarction, and renal failure [28, 29]. Particularly, a recent statement from the American Heart Association asserts that aortic stiffness is a cause rather than a consequence of hypertension in middle-aged and older individuals [30]. This statement further highlights the importance of aortic stiffening in the development of hypertension in aged people. Despite a widely held belief that aortic stiffening is associated with changes in extracellular matrix proteins and endothelial dysfunction, our recent studies demonstrated that intrinsic stiffening of aortic VSMCs, independent of VSMC proliferation and migration, is an important contributor to aortic wall stiffening both in hypertensive and aged animals [9, 10], however, the underlying molecular mechanisms remain largely unknown. The present study demonstrates for the first time that ROCK is a novel mediator of aortic VSMC stiffening in hypertension, which has never been described previously. By integrating AFM and molecular approaches, we interlinked abnormal activation of ROCK with the stiffening of aortic VSMCs in SHR. Additionally, we established that ROCK regulated VSMC stiffness through actin/SRF/myocardin signaling in aortic VSMCs of SHR. Furthermore, our study also indicated that attenuation of aortic VSMC stiffening by pharmacological inhibition, such as the inhibitors of SRF/myocardin, can serves as a promising therapeutic target to correct aortic stiffening not only in hypertension, but also in other age-related vascular diseases.

Numerous studies have shown that abnormalities in ROCK activity play an important role in cardiovascular disease and especially hypertension [11, 12]. The obvious beneficial effects from application of ROCK inhibitors have been demonstrated in numerous animal studies and human clinical trials, which support the notion that ROCKs are promising therapeutic targets for broad spectra of human diseases. However, despite the clinical importance, the molecular mechanism of ROCK on hypertension is not fully understood. It has been shown that ROCK activation plays a central role in arterial VSMC contractility, migration, differentiation and proliferation [18, 31]. In the present study, by using a direct measurement with AFM on the isolated VSMCs from stiffened aortas of hypertensive rats, we reveal a new role of ROCK in VSMC stiffness. Our results demonstrate that ROCK acts as a mediator of intrinsic VSMC mechanics for the following reasons: first, we showed ROCK activity increased in stiffer VSMCs (TA) but not in non-stiffened VSMCs (RA); second, inhibition of ROCK by Y-27632 decreased VSMC stiffness; third, VSMC stiffness exhibited a dose response to the inhibition of ROCK; fourth, removal of the inhibition of ROCK reversed the reduction of VSMC stiffness. Together, these data suggest a new role of ROCK in the pathogenesis of hypertension by contributing to VSMC stiffening, which expands its other effects demonstrated previously, including VSMC contraction and proliferation [32, 33]. We believe the current investigation is the first to identify this mechanism during the development of hypertension, which will open new avenues for treating aortic stiffness and hypertension, i.e., with pharmaceutical targets directed at the level of the VSMC itself.

Moreover, our results indicate that reduction of VSMC stiffness is a potential strategy for the reduction of aortic stiffening. Although it has been widely accepted that aortic remodeling is an important contributor to aortic stiffening in aged or hypertensive patients [34, 35], our recent studies demonstrated an unexpected yet pivotal role of VSMC stiffness in aortic stiffening [9]. Indeed, we found significant aortic remodeling evidenced by increased media layer thickness and more collagen content in the TA wall of SHR compared to WKY. However, our present study showed that chronic treatment with Y-27632 for two weeks dramatically reduced aortic stiffness without significantly reversing the aortic wall thickening and collagen deposition, implicating other arterial components, such as VSMCs, as drug-responsive contributors to the reduced aortic stiffness. Our study further supported that reduction of VSMC stiffness serves as a promising therapeutic target to correct aortic stiffness in hypertension.

Furthermore, our observations also indicated that reduction of aortic stiffening is a promising therapeutic strategy in the treatment of hypertension, particularly in the control of SBP. Despite increasing evidence supporting the causative role of aortic stiffness in the development of hypertension, drugs that directly target aortic stiffness remain limited. Data from the present study showed that inhibition of ROCK signaling by chronic Y-27632 treatment not only reduced DBP, but also caused an even greater reduction of SBP and PP in SHR. This disproportionate reduction in SBP and PP in our model is consistent with a reduction of aortic stiffness by Y-27632. Moreover, we observed that Y-27632 significantly reduced several indices of aortic stiffness, which preceded the reduction of blood pressure. This observation further supports the hypothesis that the correction of aortic stiffening is responsible for the reduction of SBP in SHR by Y-27632. These findings strongly suggested that pharmaceutical targeting of aortic stiffness correction might serve as a preventative strategy for the development of hypertension and its attendant complications.

In addition, we identified for the first time that SRF/myocardin acts downstream of ROCK signaling and plays a central role mediating VSMC stiffness in SHR TA. ROCK has been implicated in the pathogenesis of cardiovascular disease, in part by promoting ROS production, inflammation, endothelial cell damage, and VSMC contraction and proliferation [13, 18, 36, 37]. However, the molecular mechanism of ROCK in the regulation of intrinsic VSMC stiffness had not previously been identified. The present study establishes a mechanistic link between ROCK/SRF/myocardin and aortic VSMC stiffening, an important contributor to aortic vascular stiffness. SRF/myocardin is a master regulator of smooth muscle gene expression [38]. Consistent with the increase of ROCK activity and the VSMC mechanical properties, expression levels of SRF and myocardin were significantly upregulated in VSMCs from the stiffened SHR TA, and reflected a parallel alteration of downstream target genes associated with VSMC stiffness. Inhibition of ROCK by Y-27632 completely abolished the increase of SRF/myocardin and their downstream genes in SHR TA. These data collectively revealed that SRF/myocardin signaling acts as a central link between ROCK and the downstream stiffness-associated genes, thereby mediating VSMC stiffening in SHR TA. In addition to the expression of SRF/myocardin, we also observed a significant increase of the nuclear distribution of myocardin in VSMCs from SHR TA, with a concomitant shift in polymerization of G-actin into F-actin. It has been shown that SRF dependent gene transcription strongly relies on the levels of nuclear myocardin [39–41]. Increased nuclear localization of myocardin enables it to form complexes with SRF, resulting in transcription of genes that contain promoter elements that bind the SRF/myocardin complex [42]. Stimuli that result in polymerization of G-actin into F-actin release myocardin from G-actin and promote myocardin nuclear accumulation. Thus, the SRF/myocardin signaling axis also links changes in actin dynamics to SRF-dependent gene expression, promoting the VSMC stiffness.

To further determine the necessary mediating role of SRF/myocardin in VSMC stiffening, a specific inhibitor of SRF/myocardin, CCG-100602 was used to block their effects. Our data showed that inhibition of SRF/myocardin by CCG-100602 significantly decreased the VSMC stiffening in SHR TA *in vitro* and further decreased aortic stiffness *in vivo* without changing the total collagen deposition [10], which is similar to the effects of Y-27632. Moreover, by comparing the effects of two selective inhibitors, Y-27632 and CCG-100602, we further confirmed that SRF/myocardin is the specific mediator of VSMC stiffening in SHR TA, whereas ROCK acts as an upstream regulator with broad effects. There are several differences between the effects of Y-27632 and CCG-100602 observed in this study. First, CCG-100602 has no effect on ROCK activity, while Y-27632 inhibits ROCK activity. Second, although both CCG-100602 and Y-27632 suppressed expression of SRF and myocardin in SHR TA VSMCs, CCG-100602 reduced myocardin nuclear distribution and α-SMA expression only in SHR, whereas Y-27632 inhibited myocardin nuclear distribution and α-SMA in both WKY and SHR TA VSMCs. The apparently non-selective effects of Y-27632 may reflect it’s known inhibition of other serine/threonine kinases such as protein kinases A and C [43, 44]. Third, while Y-27632 reduced VSMC stiffness in both WKY and SHR TA, the effects of CCG-100602 were only observed in SHR TA, indicating the specific role of SRF/myocardin in the pathological increase of VSMC stiffness in SHR. Fourth, consistent with its non-selective effect on VSMC stiffness, Y-27632 reduced aortic stiffness in both WKY and SHR, even though the effect was reduced and delayed in WKY versus SHR; meanwhile, the effects of CCG-100602 on aortic stiffness were only observed in SHR TA[10], which further support the important role of VSMC stiffness as a determinant of aortic stiffness. These findings have important clinical relevance since one limitation of general Rho inhibitors for the treatment of cardiovascular disease is the undesirable secondary effects; for Y-27632, these include the reduction of systemic and pulmonary arterial pressures under resting baseline conditions, due to its broad effects on cytoskeletal dynamics. Selective inhibition downstream of ROCK signaling, e.g., by targeting SRF/myocardin as with CCG-100602, provides a new avenue to the development of safer therapies by specifically targeting the pathological alterations of hypertension while minimizing off-target effects.

## Conclusion

In summary, as illustrated in Fig. 7, our studies linking regulators of ROCK to VSMC stiffness in hypertension not only bridge fundamental molecular insights with pathology but also provide new ways to address targeting this pathway to limit vascular stiffening. This is important for the development of therapeutic options for vascular stiffening that occurs not only in age-related vascular disease but is also associated with vasculature abnormalities in obesity, diabetes mellitus, kidney disease, and after chemo-or radiotherapy. We expect that targeting vascular stiffness, by translating basic knowledge into effective drugs, holds great promise for the treatment of patients with cardiovascular disease.
